# Time-Lapse Into Immunofluorescence Imaging Using a Gridded Dish

**DOI:** 10.21769/BioProtoc.5606

**Published:** 2026-02-20

**Authors:** Nick Lang, Catherine G. Chu, Andrew D. Stephens

**Affiliations:** 1 Biology, University of Massachusetts Amherst, Amherst, MA, USA; 2 Molecular and Cellular Biology, University of Massachusetts Amherst, Amherst, MA, USA

**Keywords:** Live-cell imaging, Time-lapse imaging, Static imaging, Immunofluorescence, Cell biology dynamics, Nuclear dynamics, Mechanobiology, Nuclear rupture

## Abstract

Time-lapse into immunofluorescence (TL into IF) imaging combines the wealth of information acquired during live-cell imaging with ease of access for static immunofluorescence markers. In the field of mechanobiology, connecting live and static imaging to visualize cell biology dynamics is often troublesome. For instance, nuclear blebs are deformations of the nucleus that often rupture spontaneously, leading to changes in the molecular composition of the nucleus and the nuclear bleb. Current techniques to connect cellular dynamics and their downstream effects via live-cell imaging, followed by immunofluorescence, often require third-party analysis programs or stage position measurements to accurately track cells. This protocol simplifies the connection between live and static imaging by utilizing a gridded imaging dish. In our protocol, cells are plated on a dish with an engraved coordinate plane. Individual cells are then matched from when the time-lapse ends to the immunofluorescence images simply by their known coordinate location. Overall, TL into IF offers a straightforward method for connecting dynamic live-cell with static immunofluorescence imaging, in an easy and accessible tool for cell biologists.

Key features

• This protocol directly links live-cell imaging to immunofluorescence imaging.

• The only special equipment required for this protocol is gridded imaging dishes.

• This protocol does not require third-party applications.

## Graphical overview


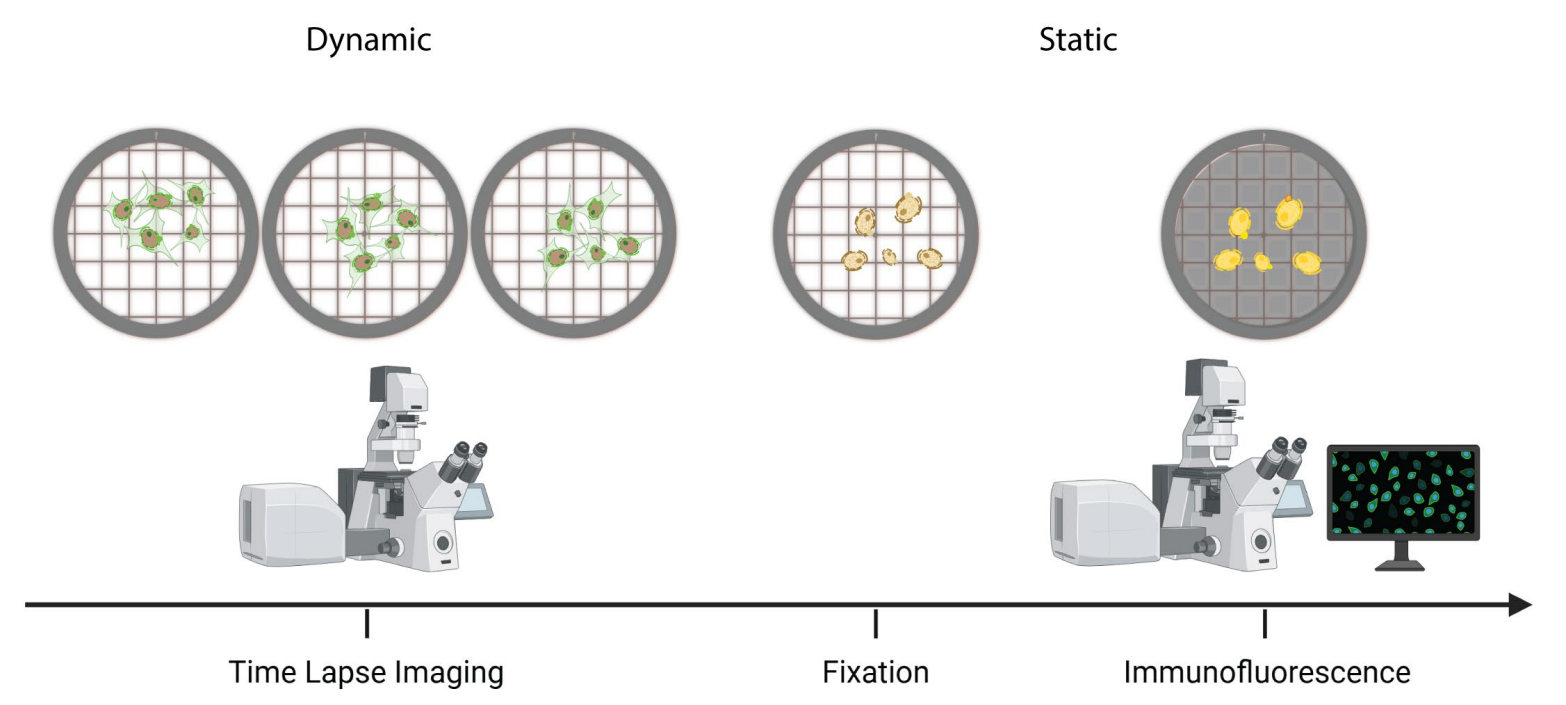


**Overview of time-lapse into immunofluorescence imaging using a gridded dish**

## Background

Both time-lapse and immunofluorescence imaging are essential to understanding cell biology dynamics and their effects. However, by performing live cell and static experiments independently of one another, information is lost. Particularly in the field of mechanobiology, it has been difficult to connect nuclear shape fluctuations and ruptures to their causes and consequences due to the disconnect between these two types of experiments. We recently overcame this barrier through the development of TL into IF [1,2]. For instance, it is known that abnormal nuclear morphology is associated with numerous human diseases, including cancer [3]. Nuclear blebs are a type of nuclear deformation that is prone to spontaneous rupture and known to cause cellular dysfunctions, including DNA damage [4–9]. However, how nuclear blebs form and their molecular composition before and after rupture has yet to be fully elucidated. It was recently noted that decreased DNA density is the best indicator of a nuclear bleb, and that loss of lamin B1 in the bleb indicates previous nuclear rupture [1,2,10,11]. Enrichment or loss of other proteins in the bleb, such as lamin A/C, emerin, and cGAS, has also demonstrated an association with nuclear rupture [1,12,13]. These experiments, as well as numerous cell biology experiments, require a direct connection between dynamic live-cell and immunofluorescence imaging to understand cellular dynamics and their downstream effects. Current methods of tracking live-cell imaging into immunofluorescence imaging often require third-party applications such as MATLAB or matching microscope stage position coordinates [14,15]. The primary advantage of our method is the elimination of these extra steps; while the use of third-party applications allows for automated tracking, our protocol does not require any external applications. Additionally, matching stage position coordinates may prove difficult if different microscopes are used for live-cell and immunofluorescence experiments. The only special equipment needed for our protocol is a gridded imaging dish (D35-14-1.5GI, Cellvis), which has a coordinate plane engraved on the glass coverslip that is visible in transmitted light. By seeding cells on the gridded dish, cells are simply matched from time-lapse imaging to immunofluorescence imaging via their location on the coordinate plane. Gridded coverslips have been used in the past for similar imaging techniques [16,17]. Our protocol decreases the complexity of the experiment by confining it to a single imaging dish, which in turn increases its accessibility and utility for cell biology.

## Materials and reagents

**Biological materials**

1. Mouse embryonic fibroblast wild-type NLS-GFP (MEF WT NLS-GFP) described in previous works [15,18,19]

**Reagents**

1. Dulbecco’s modified Eagle’s medium (DMEM) [+] 4.5 g/L glucose, L-glutamine, sodium pyruvate (Corning, catalog number: 45000-304)

2. Phosphate-buffered saline (PBS) 1× [-] calcium, magnesium (Corning, catalog number: 45000-446)

3. Characterized fetal bovine serum (FBS) (U.S.) (Cytiva HyClone^TM^, catalog number: SH3007103)

4. Penicillin-streptomycin (pen/strep) solution (Corning, catalog number: MT30002CI)

5. 16% paraformaldehyde (PFA) aqueous solution, EM grade (Electron Microscopy Sciences, catalog number: 50-980-487)

6. Bovine serum albumin (BSA) fraction V (Fisher BioReagents, catalog number: BP1605-100)

7. Triton X-100 (VWR Life Science, catalog number: 9002-93-01)

8. Tween 20 molecular biology grade (Promega, catalog number: H5152)

9. 0.25% Trypsin, 0.1% EDTA in HBSS, no calcium, magnesium, and sodium bicarbonate (Corning, catalog number: MT25053CI)

10. Primary and secondary antibodies of choice

11. Hoechst (Invitrogen, catalog number: H3570)

**Solutions**

1. DMEM medium complete (see Recipes)

2. 4% PFA solution (see Recipes)

3. Triton X-100 solution (see Recipes)

4. Tween 20 solution (see Recipes)

5. BSA solution (see Recipes)

**Recipes**

**1. DMEM medium complete**

**Table:** 

Reagent	Final concentration	Quantity or volume
DMEM	90%	500 mL
FBS	9%	50 mL
Pen/strep	1%	5.5 mL
Total	100%	555.5 mL

Thaw the frozen FBS and pen/strep in a 37 °C bead bath. Add 5.5 mL of pen/strep and 50 mL of FBS to 500 mL of DMEM. Mix by inverting. Store at 4 °C.

**2. 4% PFA solution**

**Table:** 

Reagent	Final concentration	Quantity or volume
16% PFA	25%	3 mL
PBS	75%	9 mL
Total	100%	12 mL

Add 3 mL of 16% PFA to 9 mL of PBS in a conical tube. Ensure the conical tube is covered in aluminum foil to avoid light exposure. Mix by inverting. Store at room temperature for up to one month.

*Note: Handle PFA with gloves in a fume hood and dispose of PFA in a hazardous waste container.*

**3. Triton X-100 solution**

**Table:** 

Reagent	Final concentration	Quantity or volume
Triton X-100	0.1%	400 μL
PBS	99.9%	400 mL
Total	100%	400 mL

Add 400 μL of Triton X-100 to 400 mL of PBS. Mix using a stir bar. Store at room temperature for up to one month.

**4. Tween 20 solution**

**Table:** 

Reagent	Final concentration	Quantity or volume
Tween 20	0.06%	240 μL
PBS	99.04%	400 mL
Total	100%	400 mL

Add 240 μL of Tween 20 to 400 mL of PBS. Mix using a stir bar. Store at room temperature for up to one month.

**5. BSA solution**

**Table:** 

Reagent	Final concentration	Quantity or volume
BSA	2%	500 mg
PBS	98%	25 mL
Total	100%	25 mL

Add 500 mg of BSA to 25 mL of PBS. Mix by inverting. The BSA solution can be stored at 4 °C for up to one week.

**Laboratory supplies**

1. 60 mm surface-treated tissue culture dishes (Fisher, catalog number: FB012921)

2. 35 mm glass bottom dish with 14 mm micro-well #1.5 gridded cover glass (Cellvis, catalog number: D35-14-1.5GI)

3. 15 mL conical tubes (VWR International, catalog number: 470225-000)

4. 50 mL conical tubes (VWR International, catalog number: 470225-004)

5. 2 mL serological pipettes (Fisher, catalog number: 13-678-11C)

6. 5 mL serological pipettes (Fisher, catalog number: 13-678-11D)

7. 10 mL serological pipettes (Fisher, catalog number: 13-678-11E)

8. 2 μL pipette tips (Gilson, catalog number: 76178-284)

9. 20 μL pipette tips (Gilson, catalog number: 76178-282)

10. 1,000 μL pipette tips (Gilson, catalog number: 76180-360)

11. Parafilm (Amcor, catalog number: 13-374-5)

## Equipment

1. Incubator (Heracell VIOS 160i Tri-Gas CO_2_ Incubator) (Thermo Scientific, catalog number: 51033720)

2. Pipet-Aid XP2 (Drummond Scientific Company, catalog number: 4-000-501)

3. Nutating mixer (VWR International, catalog number: 82007-202)

4. Light microscope [Nikon Instruments Ti-2E microscope, Orca Fusion Gen III Camera, Lumencor Aura III light engine, Perfect Focus System, TMC CleanBench air table, with 40× air objective (N.A 0.75, W.D. 0.66, MRH00401) or use with Crest V3 Spinning Disk Confocal]

5. Stage heater (Okolab Stage Heater, model: H401-T-Controller)

6. Stage top incubator (Okolab Stage Top Incubator, model: H301)

7. Refrigerator (4 °C)

## Procedure

**A. Preparing the gridded dish**

1. Plate MEF WT NLS-GFP in 37 °C DMEM medium complete (see Recipe 1) on a single-well gridded dish. Incubate overnight at 37 °C with 5.0% CO_2_ to allow cells to adhere.

*Note: Plate cells to reach 50%–70% confluency on the day of time-lapse imaging. Take into account drug incubation times.*

**B. Time-lapse imaging**

1. Allow the microscope stage to reach 37 °C and 5.0% CO_2_.

2. Transfer the gridded dish from the incubator to the microscope.

**Figure 1.**
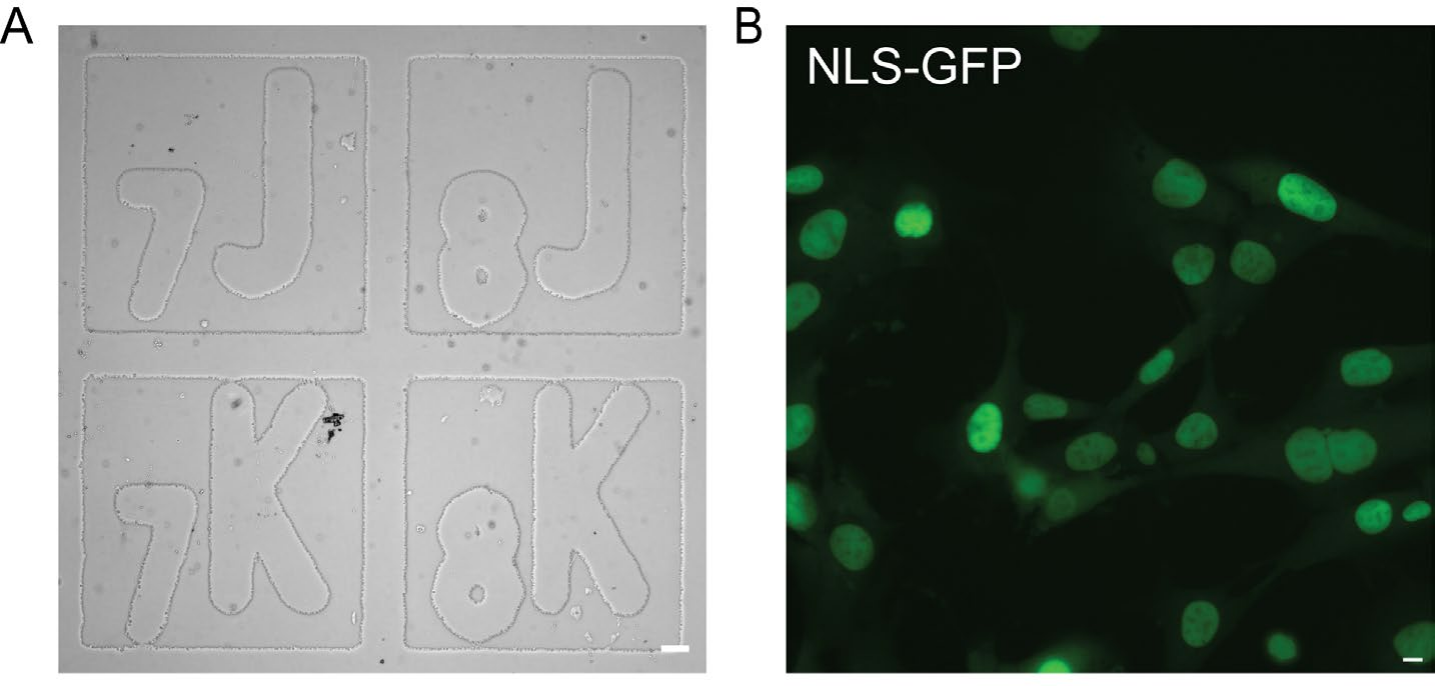
Gridded dish preparation for time-lapse imaging. (A) Representative image of a gridded dish in a coordinate-oriented right side up under transmitted light (scale bar = 50 μm). (B) Representative image of a field of view of MEF WT NLS-GFP cells in a coordinate under fluorescein isothiocyanate (FITC) light (excitation 498 nm, emission 517 nm) (scale bar = 10 μm).

*Note: Orient the dish so that the grid is right side up. Check the dish under transmitted light to ensure the dish is oriented correctly (Figure 1A). With a marker, mark the bottom of the dish so the orientation can be matched during immunofluorescence imaging.*

3. Select fields of view with 70% confluency (Figure 1B). Note the coordinate for each field of view, e.g., A7, CS, etc.

*Note: Select adjacent coordinates to make cell matching easier. Ensure that the fields of view have no overlapping cells.*

4. Run the time-lapse according to standard protocol.

**Critical:** Perform the following steps as quickly as possible after the time-lapse ends to minimize cell movement.

5. Remove cells from the microscope and transfer them back to the cell culture hood. Replace DMEM with 3 mL of PBS.

6. To fix cells, prepare 4% PFA (see Recipe 2) in a 15 mL conical tube in the fume hood. From this point forward, keep the dish covered with foil to prevent light exposure.

7. Remove PBS and add 3 mL of 4% PFA to the gridded dish. Dispose of PFA in a hazardous waste container. Cover in foil and let sit for 15 min at room temperature.

**Caution:** Wash gently to minimize cell movement.

8. Remove 4% PFA and wash with 3 mL of PBS for 5 min. Repeat two times.

**Pause point:** Fixed cells can be stored in 3 mL of PBS at 4 °C for up to one month before the protocol is resumed. Wrap cells in parafilm to prevent evaporation. Incomplete fixation of cells may result in shorter storage times.

**C. Immunofluorescence**

1. Remove PBS and add 3 mL of Triton X (see Recipe 3). Let it sit for 15 min at room temperature.

2. Remove Triton X and add 3 mL of Tween 20 (see Recipe 4). Let it sit for 5 min at room temperature.

3. Remove Tween 20 and wash with 3 mL of PBS for 5 min. Repeat two times.

4. To block cells, prepare BSA (see Recipe 5) in a 50 mL conical tube. Allow to dissolve completely at 4 °C.

5. Remove PBS from the dish and add 3 mL of BSA. Let it sit for 1 h at room temperature.

6. Remove BSA and add 1,000 μL of primary antibody solution according to the standard protocol. Wrap in parafilm and let it sit overnight at 4 °C.

*Note: Pipette the antibody solution directly onto the cells in the center portion of the coverslip, not into the outer plastic ring.*

7. Remove the primary antibody solution and wash with 3 mL of PBS for 5 min. Repeat two times.

8. Remove PBS and add 1,000 μL of secondary antibody solution according to the standard protocol. Let it sit on a nutating mixer for 1 h at room temperature.

*Note: Pipette the antibody solution directly onto the cells in the center portion of the coverslip, not into the outer plastic ring.*

9. Remove the secondary antibody solution and wash with 3 mL of PBS for 5 min. Repeat two times.

10. Remove PBS and add 1,000 μL of Hoechst DNA stain solution according to the standard protocol. Let it sit at room temperature for 15 min.

*Note: Pipette the staining solution directly onto the cells in the center portion of the coverslip, not into the outer plastic ring.*

11. Remove the DNA stain solution and wash with 3 mL of PBS for 5 min. Repeat two times.

12. Replace PBS with 3 mL of fresh PBS.

**D. Locating cells via immunofluorescence imaging**

**Figure 2.**
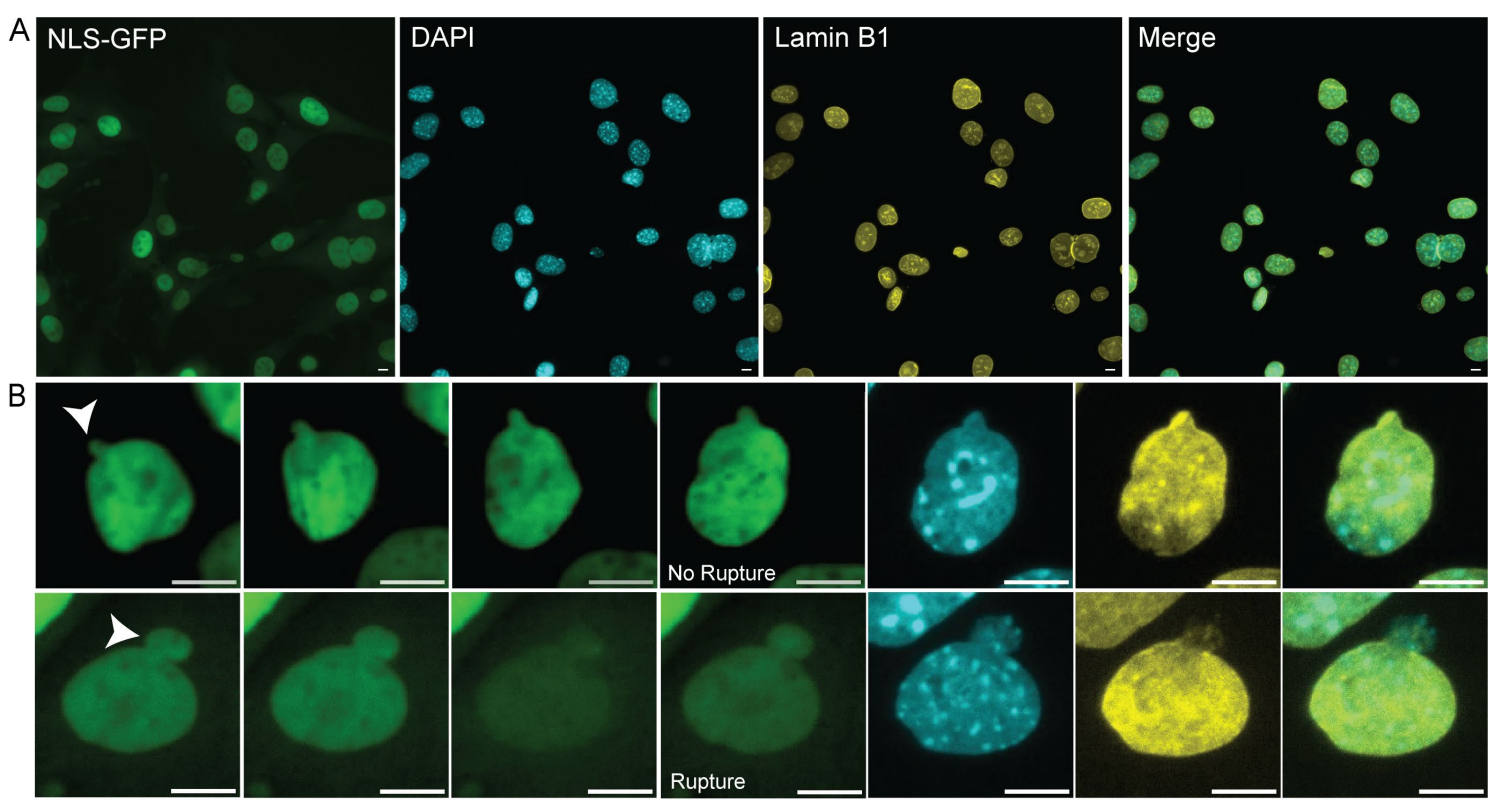
Matching time-lapse field of view during immunofluorescence imaging. (A) Representative image of the last frame of a time-lapse field of view under fluorescein isothiocyanate (FITC) light (excitation 498 nm, emission 517 nm). Matching immunofluorescence images stained with Hoechst, lamin B1, and merged. Scale bars, 10 μm. (B) Top row: example images from time-lapse imaging of a blebbed unruptured nucleus and subsequent immunofluorescence (NLS-GFP images from time 0, 1, 2, and 3 h; white arrow denotes bleb). Bottom row: example images from time-lapse imaging of a blebbed ruptured nucleus and subsequent immunofluorescence (NLS-GFP images from time 0, 2, 4, and 16 min; white arrow denotes bleb). Scale bars, 10 μm.

1. Place the dish onto the microscope in the same orientation as during time-lapse imaging. Use the previously drawn mark as a guide.

2. Open time-lapse images in the desired software and select the last frame of each field of view to use as a visual reference.

3. Locate the recorded coordinates from fields of view during the time-lapse in transmitted light and adjust so that the cells match the last frame in the time-lapse (Figure 2).

4. Locate all of the matching fields of view from the time-lapse. Image matching fields of view according to the standard protocol. Name each immunofluorescence field of view to match its time-lapse counterpart.

## Validation of protocol

This protocol has been used and validated in the following research articles:

• Chu et al. [1]. Lamin B loss in nuclear blebs is rupture dependent while increased DNA damage is rupture independent. Journal of Cell Science (Figure 1A).

• Bunner et al. [2]. Decreased DNA density is a better indicator of a nuclear bleb than lamin B loss. Journal of Cell Science.

## General notes and troubleshooting

**General notes**

1. Depending on the length of the time-lapse, this protocol likely takes two days. To complete the protocol in one day, primary antibodies can be incubated at 37 °C for 2 h or according to the standard protocol. The rest of the protocol can then be completed as normal, following three washes with PBS.

**Troubleshooting**

**Problem 1:** Cells have either moved or disappeared from their original position during immunofluorescence imaging.

Possible causes: Waiting too long to fix after the time-lapse ends; harsh washing.

Solutions: Fix the cells as soon as the time-lapse ends. Gently wash the cells by pipetting liquids in and out of the outer plastic ring rather than directly onto the cells in the center.

## References

[1] Chu C. G., Lang N., Walsh E., Zheng M. D., Manning G., Shalin K., Cunha L. M., Faucon K. E., Kam N., Folan S. N., .(2025). Lamin B loss in nuclear blebs is rupture dependent whereas increased DNA damage is rupture independent. J Cell Sci. 138(21): e263945. https://doi.org/10.1242/jcs.263945 PMC12669966 41047936

[2] Bunner S., Prince K., Pujadas Liwag E. M., Eskndir N., Srikrishna K., McCarthy A. A., Kuklinski A., Jackson O., Pellegrino P., Jagtap S., .(2024). Decreased DNA density is a better indicator of a nuclear bleb than lamin B loss. J Cell Sci. 138(3): e262082. https://doi.org/10.1242/jcs.262082 PMC11883270 39501901

[3] Kalukula Y., Stephens A. D., Lammerding J. and Gabriele S. (2022). Mechanics and functional consequences of nuclear deformations. Nat Rev Mol Cell Biol. 23(9): 583 602 602. 10.1038/s41580-022-00480-z 35513718 PMC9902167

[4] Denais C. M., Gilbert R. M., Isermann P., McGregor A. L., M. te Lindert, Weigelin B., Davidson P. M., Friedl P., Wolf K., Lammerding J., .(2016). Nuclear envelope rupture and repair during cancer cell migration. Science. 352(6283): 353 358 358. 10.1126/science.aad7297 27013428 PMC4833568

[5] Xia Y., Ivanovska I. L., Zhu K., Smith L., Irianto J., Pfeifer C. R., Alvey C. M., Ji J., Liu D., Cho S., .(2018). Nuclear rupture at sites of high curvature compromises retention of DNA repair factors. J Cell Biol. 217(11): 3796 3808 3808. 10.1083/jcb.201711161 30171044 PMC6219729

[6] Shah P., Hobson C. M., Cheng S., Colville M. J., Paszek M. J., Superfine R. and Lammerding J. (2021). Nuclear Deformation Causes DNA Damage by Increasing Replication Stress. Curr Biol. 31(4): 753 765 765 .e6. 10.1016/j.cub .2020.11.037 33326770 PMC7904640

[7] Pho M., Berrada Y., Gunda A., Lavallee A., Chiu K., Padam A., Currey M. L. and Stephens A. D. (2024). Actin contraction controls nuclear blebbing and rupture independent of actin confinement. Mol Biol Cell. 35(2): ee23–07–0292. https://doi.org/10.1091/mbc.e23-07-0292 PMC10881147 38088876

[8] Raab M., Gentili M., de Belly H., Thiam H. R., Vargas P., Jimenez A. J., Lautenschlaeger F., Voituriez R., Lennon-Duménil A. M., Manel N., .(2016). ESCRT III repairs nuclear envelope ruptures during cell migration to limit DNA damage and cell death. Science. 352(6283): 359 362 362. 10.1126/science.aad7611 27013426

[9] Stephens A. D., Liu P. Z., Kandula V., Chen H., Almassalha L. M., Herman C., Backman V., T. O’Halloran, Adam S. A., Goldman R. D., .(2019). Physicochemical mechanotransduction alters nuclear shape and mechanics via heterochromatin formation. Mol Biol Cell. 30(17): 2320 2330 2330. 10.1091/mbc.e19-05-0286 31365328 PMC6743459

[10] Pujadas Liwag E. M., Acosta N., Almassalha L. M., Y. Su(., Gong R., Kanemaki M. T., Stephens A. D. and Backman V. (2025). Nuclear blebs are associated with destabilized chromatin-packing domains. J Cell Sci. 138(3): e262161. https://doi.org/10.1242/jcs.262161 PMC11883274 39878045

[11] Stephens A. D., Banigan E. J. and Marko J. F. (2017). Separate roles for chromatin and lamins in nuclear mechanics. Nucleus. 9(1): 119 124 124. 10.1080/19491034.2017 .1414118 29227210 PMC5973264

[12] Young A. M., Gunn A. L. and Hatch E. M. (2020). BAF facilitates interphase nuclear membrane repair through recruitment of nuclear transmembrane proteins. Mol Biol Cell. 31(15): 1551 1560 1560. 10.1091/mbc.e20-01-0009 32459568 PMC7521799

[13] Halfmann C. T., Sears R. M., Katiyar A., Busselman B. W., Aman L. K., Zhang Q., O’Bryan C. S., Angelini T. E., Lele T. P., Roux K. J., .(2019). Repair of nuclear ruptures requires barrier-to-autointegration factor. J Cell Biol. 218(7): 2136 2149 2149. 10.1083/jcb.201901116 31147383 PMC6605789

[14] Teague S., Primavera G., Chen B., Liu Z. Y., Yao L., Freeburne E., Khan H., Jo K., Johnson C., Heemskerk I., .(2024). Time-integrated BMP signaling determines fate in a stem cell model for early human development. Nat Commun. 15(1): 1471 10.1038/s41467-024-45719-9 38368368 PMC10874454

[15] Shimi T., Pfleghaar K., Kojima S., Pack C. G., Solovei I., Goldman A. E., Adam S. A., Shumaker D. K., Kinjo M., Cremer T., .(2008). The A- and B-type nuclear lamin networks: microdomains involved in chromatin organization and transcription. Genes Dev. 22(24): 3409 3421 3421. 10.1101/gad.1735208 19141474 PMC2607069

[16] van Rijnsoever C., Oorschot V. and Klumperman J. (2008). Correlative light-electron microscopy(CLEM) combining live-cell imaging and immunolabeling of ultrathin cryosections. Nat Methods. 5(11): 973 980 980. 10.1038/nmeth.1263 18974735

[17] Asakawa H., Hiraoka Y. and Haraguchi T. (2014). A method of correlative light and electron microscopy for yeast cells. Micron. 61: 53 61 61. 10.1016/j.micron .2014.02.007 24792447

[18] Stephens A. D., Liu P. Z., Banigan E. J., Almassalha L. M., Backman V., Adam S. A., Goldman R. D. and Marko J. F. (2018). Chromatin histone modifications and rigidity affect nuclear morphology independent of lamins. Mol Biol Cell. 29(2): 220 233 233. 10.1091/mbc.e17-06-0410 29142071 PMC5909933

[19] Vahabikashi A., Sivagurunathan S., Nicdao F. A. S., Han Y. L., Park C. Y., Kittisopikul M., Wong X., Tran J. R., Gundersen G. G., Reddy K. L., .(2022). Nuclear lamin isoforms differentially contribute to LINC complex-dependent nucleocytoskeletal coupling and whole-cell mechanics. Proc Natl Acad Sci USA. 119(17): e2121816119. https://doi.org/10.1073/pnas.2121816119 PMC9170021 35439057

